# Association Between Lipophilic Beta-Blockers and Depression in Diabetic Patients on Chronic Dialysis

**DOI:** 10.1177/11795514221119446

**Published:** 2022-08-29

**Authors:** Robin Lengton, Robbert W Schouten, Els Nadort, Elisabeth FC van Rossum, Friedo W Dekker, Carl EH Siegert, Ellen K Hoogeveen

**Affiliations:** 1 Department of Clinical Epidemiology, Leiden University Medical Center, Leiden, The Netherlands; 2 Department of Nephrology, OLVG hospital, Amsterdam, The Netherlands; 3 Department of Psychiatry, OLVG hospital, Amsterdam, The Netherlands; 4 Department of Internal Medicine, Division of Endocrinology, Erasmus University Medical Center Rotterdam, Rotterdam, The Netherlands; 5 Department of Nephrology, Jeroen Bosch Hospital, Den Bosch, The Netherlands

**Keywords:** Beta-blockers, depressive symptoms, diabetes, dialysis, diabetic kidney disease

## Abstract

**Background:**

Depression is associated with lower quality of life and increased risk of mortality. The prevalence of depression in chronic dialysis patients, as well as in patients with diabetes, is more than 20%. It is debated whether use of beta-blockers increases the risk of depression. Therefore, we examined in chronic dialysis patients with and without diabetes, the association between beta-blockers and depressive symptoms.

**Methods:**

Data were collected from the DIVERS-I study, a multicentre prospective cohort among chronic dialysis patients in the Netherlands. Depressive symptoms were assessed with the Beck Depression Inventory (BDI-II). We defined depressive symptoms as a BDI-II score ⩾16. The cross-sectional association at baseline between depressive symptoms and beta-blocker use in chronic dialysis patients, was studied by multivariable logistic regression adjusted for potential confounders.

**Results:**

We included 684 chronic dialysis patients, of whom 43% had diabetes mellitus, and 57% used a beta-blocker of which 97% were lipophilic. After multivariable adjustment, the OR (95% CI) for depressive symptoms in patients with compared to without diabetes was 1.41 (1.00-1.98), and in beta-blocker users compared to non-users 1.12 (0.80-1.56), respectively. Dialysis patients with diabetes and beta-blocker use compared to those without diabetes and not using beta-blockers had an OR of 1.73 (1.12-2.69) for depressive symptoms. The association was stronger in dialysis patients with diabetes and lipophilic beta-blocker use with an OR of 1.77 (1.14-2.74).

**Conclusions:**

We found a possible association between lipophilic beta-blocker use and depressive symptoms in chronic dialysis patients with diabetes.

## Introduction

The prevalence of depressive symptoms in dialysis patients, being more than 37%, is exceptionally high.^1^ It is the most common mental health condition, with 15% to 23% of the dialysis patients having symptoms of depression.^1,2^ Depressive symptoms are a major burden to the individual dialysis patient, causing decreased quality of life, less adherence to dialysis prescription and lifestyle advice, and increased hospitalisation and mortality.^3-6^ To improve quality of life and life expectancy of chronic dialysis patients, identification of high risk patients and modifiable risk factors for depression is important.

Type 2 diabetes (T2D) is highly prevalent (40%) as co-morbidity among dialysis patients and an important risk factor of depression.^7,8^ There is strong evidence that T2D and depression are interconnected through a vicious, mutually reinforcing cycle of adverse physiological adaptations. Shared biological mechanisms may explain this relation at different levels, from genes and peripheral endocrine, immuno-inﬂammatory and metabolic mechanisms to the brain.^9,10^

Dialysis patients with diabetes are frequently prescribed beta-blockers for treatment of hypertension or increased cardiovascular risk.^11^ However, beta-blockers, especially those with lipophilic properties, may have adverse neuropsychological side effects.^12,13^ Lipophilic beta-blockers are able to pass the blood-brain-barrier which may cause central nervous system side-effects such as depressive symptoms.^14,15^ Studies among patients with hypertension, heart failure or myocardial infarction (MI), showed an association between depressive symptoms and the use of beta-blockers.^14,16,17^ In addition, higher Beck et al Depression Inventory (BDI-II) scores, an inventory for measuring depression, were observed in patients using a higher dosage of beta-blockers or with long-term use.^14,17^ To our knowledge, no studies have been performed in dialysis patients.

Since beta-blocker use is a potential modifiable risk factor, we investigated the risk of depressive symptoms in diabetic chronic dialysis patients with and without beta-blocker use. These results may inform future guidelines about prevention of depressive symptoms and increase awareness of clinicians about the possible adverse effects of beta-blockers in chronic dialysis patients.

## Materials and Methods

### Study design and population

The DIVERS-I study is a prospective multicentre cohort study of 684 chronic dialysis patients, as previously described in detail.^18^ In brief, inclusion criteria were: ⩾18 years of age, undergoing dialysis treatment for at least 90 days (haemodialysis (HD) or peritoneal dialysis (PD)) and being able to fill in the questionnaires (available in 4 languages, help was provided if necessary). Patients were excluded if they were not able to fill in the questionnaire due to cognitive impairment or language barrier, or being physically too ill to participate. Patients were recruited in 1 of 10 dialysis clinics in Amsterdam and The Hague in the Netherlands. Inclusion ran from June 2012 until December 2013 for prevalent patients, incident patients were included until October 2014. All patients were treated by their nephrologist in accordance with the treatment guidelines of the Dutch Federation of Nephrology, guidelines partly based on the K/DOQI and EBPG guidelines.^19^

The DIVERS-I study was conducted in accordance with the Declaration of Helsinki and was approved by the medical ethics committee of the VU university medical centre (approval number: 2010/064). Written informed consent was obtained from all patients prior to study inclusion.

### Data collection

Information on demographic and clinical variables were collected from electronic medical records: age, sex, dialysis modality (HD or PD), dialysis vintage, body mass index (BMI), diabetes mellitus type 1 (T1D) or T2D (yes/no), history of cardiovascular disease (CVD: yes/no), comorbidity (scored according to Davies et al comorbidity index, resulting in no, intermediate or severe comorbidity), anti-depressant use (yes/no) and the primary cause of kidney disease (classified according to the coding system of the European Renal Association-European Dialysis and Transplant Association (ERA-EDTA)).^20,21^ T2D is considered present in case of a self-reported physician’s diagnosis and/or use of glucose lowering drugs. CVD is considered to be present in case of a history of MI/acute coronary syndrome, percutaneous transluminal coronary angioplasty (PTCA), coronary artery bypass surgery (CBAG), heart failure, peripheral arterial disease or cerebrovascular accident (CVA). Medication is coded according to the Anatomical Therapeutic Chemical (ATC) Classification System. Weekly KT/V for HD and PD was collected from medical records.^22^ Data on ethnicity (distinction was made between native and immigrant, based on country of birth), current smoking of cigarettes (yes/no), alcohol consumption (yes/no) and educational level (high vs low) was collected via a self-reported questionnaire.

### Assessment of depression

The BDI-II was used to measure symptoms of depression.^17^ The BDI-II consists of 21 questions, which rate severity on a scale of 0 to 3, with a total score of 63. Cronbach’s Alpha analysis showed an alpha of .89, which indicates a high level of internal consistency for the scale of the questionnaire. We define depressive symptoms as BDI-II score of ⩾16.^17,23^

### Beta-blocker use

Data on the usage of beta-blockers were collected from medical records, defined by receipt of single prescription or longer term use. The following beta-blockers were included: Bisoprolol, Carvedilol, Labetalol, Metoprolol and Nebivolol, classified as lipophilic, as well as Atenolol and Sotalol, classified as hydrophilic.

### Data analysis

Baseline characteristics are presented for all 684 patients, and stratified for diabetes (including T1D and T2D) and beta-blocker use, as mean ± SD, median and interquartile range or number and percentage, depending on the underlying distribution. Difference 95% confidence interval were calculated using online CI calculators.^24,25^

We used multiple imputation for the main analyses on all confounders and the sub-questions of the BDI-II to avoid bias and maintain power, including all relevant baseline variables and the outcome in the model. The following number of data were missing: age (n = 1), smoking (n = 93), alcohol consumption (n = 86), ethnicity (n = 57), educational level (n = 98) and dialysis vintage (n = 1). We used 10 imputations. Sensitivity analyses were performed on mean substitution data and complete cases.

Logistic regression was used to study the cross-sectional association at baseline between the covariates and depressive symptoms. The covariates include age, sex, education, ethnicity, smoking, alcohol use, dialysis vintage, CVD, hypertension, diabetes, beta-blocker use and lipophilic beta-blocker use. Results are presented crude and adjusted for pre-defined potential confounders including age, sex, current smoking, alcohol consumption, ethnicity, educational level, dialysis vintage, diabetes and CVD (full model). However, it is thought that CVD might be one of the major factors in the causal pathway between diabetes and depressive symptoms, but this relation is complex. In contrast, CVD is also a proxy for beta-blocker use, and may therefore cause confounding by indication. For this reason CVD is included in the model.

To examine the joint and separate effects of the risk factors diabetes and beta-blockers on depressive symptoms, the relative excess risk due to interaction (RERI) was calculated from the odds ratios (ORs) obtained by logistic regression analysis. This approach explores if the effect when both risk factors are jointly presented differs compared to the sum of both risk factors presented separately. A RERI value of 0 means no interaction; above 0 means positive additive interaction and below 0 mean negative additive interaction.^26^ The biologic interaction was also calculated by the synergy index (S), which describes the ratio of the joint effect (presence of both risk factors) to the sum of the effects (presence of each risk factor in the absence of the other).^27-29^ When *S* = 1, there is no biologic interaction; *S* < 1 measures antagonism and *S* > 1 means synergy/interaction.^30^ The model was adjusted for sex, age and ethnicity. Calculations of the 95% CI are described elsewhere.^31^

Sensitivity analyses were first performed after mean substitution according to the manual for missing BDI-II items and second on complete cases.^32^ The BDI-II scores could only be calculated if all 21 questions were filled in. In total, the BDI-II score was missing for 151 (22.1%) patients. Mean substitution according to the manual was used to compensate for incomplete questionnaires, in case not all 21 question were filled in. The computed BDI-II scores were calculated by dividing the original BDI-II score by the amount of questions filled in, multiplied by the total amount of questions (21). In this way the BDI-II score range was maintained. For the sensitivity analysis we repeated the main analysis using a cut-off value of ⩾16 on the BDI-II as the outcome with the imputed and complete case data.

All statistical analysis were performed using SPSS statistical software, version 24 (IBM Corp., Armonk, NY, USA).^33^

## Results

### Baseline characteristics

Baseline characteristics of all patients (N = 684) of the DIVERS-I study are presented in Table 1. In total, 291 (43%) patients had diabetes and 387 (57%) patients used beta-blockers of whom 97% were lipophilic beta-blockers. Patients with versus without diabetes had shorter dialysis vintage; more intermediate and severe Davies comorbidity score; had more often hypertension; were more often users of beta-blockers, RAS-inhibitors and diuretics; and had a higher BDI-II score. Beta-blocker users versus non-users had more often diabetes, hypertension and a more severe Davies comorbidity score.

**Table 1.** Baseline characteristics of 684 chronic dialysis patients of the DIVERS-I study stratified for diabetes and the use of beta-blockers.

Baseline variables	All patients N = 684	Diabetes^a^		Beta-blockers^b^	
		Yes N = 291	No N = 393	Users N = 387	Non-users N = 297
Age, y	64.5 ± 15.3	67.2 ± 12.0	62.5 ± 17.1	65.0 ± 14.6	63.7 ± 16.14
Men, n (%)	422 (61.7)	175 (60.1)	247 (62.8)	247 (63.8)	175 (58.9)
Married, yes (%)	316 (52.4)	129 (49.8)	187 (54.4)	174 (52.4)	142 (52.4)
Children, yes (%)	474 (78.0)	216 (82.1)	258 (74.8)	266 (79.2)	208 (76.5)
Educational level, low (%)	332 (56.7)	167 (65.5)	165 (49.8)	185 (56.7)	147 (56.5)
Race, n (%)					
Caucasian	366 (58.1)	129 (48.3)	237 (65.3)	197 (55.6)	169 (61.2)
Asian	80 (12.7)	35 (13.1)	45 (12.4)	43 (12.1)	37 (13.4)
Black	184 (29.2)	103 (38.6)	81 (22.3)	114 (32.2)	70 (25.4)
Immigrant status, n (%)					
Native	327 (52.2)	120 (45.1)	207 (57.3)	175 (49.7)	152 (55.3)
Immigrant	300 (47.8)	146 (54.9)	154 (42.7)	177 (50.3)	123 (44.7)
Unemployement,^c^ n (%)	534 (88.6)	239 (91.9)	295 (86.0)	297 (89.7)	237 (87.1)
Smoking, current (%)	108 (18.3)	41 (16.1)	67 (19.9)	69 (21.2)	39 (14.7)
Alcohol use, yes (%)	161 (26.9)	54 (20.8)	107 (31.6)	89 (27.1)	72 (26.8)
BDI-II score	12.9 ± 9.6	14.3 ± 10.4	11.9 ± 8.8	13.4 ± 9.7	12.2 ± 9.4
Health related quality of life (SF-12)					
Physical component summary score	38.1 ± 11.1	36.9 ± 11.0	39.0 ± 11.2	38.4 ± 11.1	37.7 ± 11.1
Mental component summary score	48.9 ± 10.8	47.6 ± 11.1	49.8 ± 10.6	48.3 ± 11.1	49.5 ± 10.6
Dialysis modality, HD (%)	600 (87.7)	262 (90.0)	338 (86.0)	337 (87.1)	263 (88.6)
Dialysis vintage, months	12 (12-45)	11 (4-41)	13 (4-49.8)	11 (4-45)	14.5 (5-48.8)
Residual diuresis, ⩾ 100 ml/24h (%)	487 (71.2)	210 (72.2)	277 (70.5)	286 (73.9)	201 (67.7)
KT/V (urea) HD, weekly	4.2 ± 1.1	4.2 ± 1.2	4.2 ± 1.1	4.1 ± 1.1	4.3 ± 1.2
KT/V (urea) PD, weekly	2.2 ± 0.7	2.2 ± 0.8	2.2 ± 0.6	2.3 ± 0.7	2.0 ± 0.6
Primary cause of renal failure, n (%)					
Diabetes	155 (24.4)	155 (56.4)	0	110 (30.3)	45 (16.6)
Glomerulonephritis	70 (11.0)	18 (6.5)	52 (14.5)	41 (11.3)	29 (10.7)
Renal vascular disease	163 (25.7)	54 (19.6)	109 (30.4)	97 (26.7)	66 (24.4)
Other	246 (38.8)	48 (17.5)	198 (55.2)	115 (31.7)	131 (48.3)
CVD, yes (%)	308 (45.0)	175 (60.1)	133 (33.8)	212 (54.8)	96 (32.3)
Davies comorbidity^d^, n (%)					
No	182 (27.1)	2 (0.7)	181 (46.9)	78 (20.4)	104 (36.0)
Intermediate	370 (55.1)	186 (64.8)	184 (47.9)	220 (57.6)	150 (51.9)
Severe	119 (17.7)	99 (34.5)	20 (5.2)	84 (22.0)	35 (12.1)
BMI, kg/m^2^	27.0 ± 6.2	29.0 ± 7.0	25.5 ± 5.0	27.4 ± 6.6	26.5 ± 5.6
Diabetes^a^, yes (%)	291 (42.5)	291 (100)	0	190 (49.1)	101 (34.0)
Treated for diabetes, yes (%)	224 (32.7)	224 (77.0)	0	151 (79.5)	73 (72.3)
Oral medication^e^	57 (8.3)	57 (19.6)	0	30 (15.8)	27 (26.7)
Insulin medication^f^	184 (26.9)	184 (63.2)	0	131 (68.9)	53 (52.5)
Blood pressure					
Systolic blood pressure before HD	146.8 ± 24.8	149.1 ± 27.3	144.9 ± 22.5	148.3 ± 24.8	144.7 ± 24.6
Diastolic blood pressure before HD	72.4 ± 16.1	69.3 ± 15.7	74.8 ± 16.0	71.5 ± 15.6	73.6 ± 16.6
Systolic blood pressure PD	141.1 ± 22.4	146.2 ± 23.2	138.4 ± 21.7	143.8 ± 22.5	137.3 ± 22.1
Diastolic blood pressure PD	80.3 ± 13.9	78.9 ± 12.4	81.1 ± 14.7	81.0 ± 14.6	79.3 ± 13.0
Hypertension,^g^ yes (%)	436 (63.7)	211 (72.5)	225 (57.3)	275 (71.1)	161 (54.2)
Anti-hypertensives, yes (%)	502 (73.4)	230 (79.0)	272 (69.2)	319 (82.4)	183 (61.6)
Beta-blockers^a^	387 (56.6)	190 (65.3)	197 (50.1)	387 (100)	0
Alpha-blockers^h^	59 (8.6)	26 (8.9)	33 (8.4)	46 (11.9)	13 (4.4)
RAS-inhibitors^i^	288 (42.1)	136 (46.7)	152 (38.7)	191 (49.4)	97 (32.7)
Diuretics^j^	355 (51.9)	183 (62.9)	172 (43.8)	231 (59.7)	124 (41.8)
Calcium-antagonists^k^	230 (33.6)	97 (33.3)	133 (33.8)	150 (38.8)	80 (26.9)
Anti-depressants,^l^ yes (%)	65 (9.5)	39 (13.4)	26 (6.6)	36 (9.3)	29 (9.8)

Abbreviations: BDI, Beck Depression Inventory (1979); BMI, body mass index; CVD, cardiovascular disease; HD, haemodialysis; KT/V, a number used to quantify haemodialysis and peritoneal dialysis treatment adequacy; PD, peritoneal dialysis; RAS, renin–angiotensin system.

a Diabetes mellitus was considered present in case of a self-reported physician’s diagnosis and/or use of glucose lowering drugs.

b Beta-adrenergic blocking agents: Anatomical Therapeutic Chemical Classification System (ATC) codes C07AA07, C07AB02, C07AB03, C07AB07, C07AB12, C07AG01 and C07AG02.

c No (paid) job.

d Based on the presence or absence of 7 comorbidities (without primary kidney disease as comorbid disease). Low risk is classified as having no comorbid conditions; intermediate as having 1 or 2 comorbid conditions and severe as having 3 or more comorbid diseases.

e Blood glucose lowering drugs: ATC codes A10BB09, A10BH02, A10BB03, A10BH05 and A10BX02.

f Insulins and analogues: ATC codes A10AB01, A10AB05, A10AB06, A10AC01, A10AD01, A10AD05, A10AE04, A10AE05, and A10AE56.

g Physician diagnosed.

h Alpha-adrenoreceptor antagonists: ATC codes C02CA04 and C02DC01.

i Agents acting on the renin–angiotensin system: ATC codes C09AA02, C09AA03, C09AA04, C09AA05, C09AA06 C09AA09, C09CA01, C09CA03, C09CA04, C09CA06, C09CA07, C09CA08, C09DA04 and C09DB02.

j Diuretics: ATC codes C03CA01, C03CA02, C03DA04, C03DB02 and C09DA0.

k Calcium channel blockers: ATC codes C08CA01, C08CA04, C08CA05, C08CA12, C08CA13 and C09DB02.

l Self-reported.

### BDI-II scores and risk of depressive symptoms

In total, from 533 out of 684 (78%) patients the complete BDI-II score was known with a mean ± SD of 12.9 ± 9.6 and an overall prevalence of depressive symptoms (BDI-II ⩾ 16) of 31%. The BDI-II score in patients with (N = 223) versus without (N = 310) diabetes was 14.3 ± 10.4 and 11.9 ± 8.8 (difference 95% CI: 0.81-4.09), respectively. The prevalence of depressive symptoms in patients with diabetes was 37% compared to 26% in patients without diabetes (difference 95% CI: 3%-19%). Stratification for beta-blocker use resulted in a BDI-II score of 13.4 ± 9.7 for users (N = 300) and 12.2 ± 9.4 for non-users (N = 233) (difference 95% CI: −0.42 to 2.86), with a prevalence of depressive symptoms of 34% in users compared to 27% in the non-users (difference 95% CI: −1%to 15%). In total, 49% of the beta-blocker users had diabetes from whom 41% had depressive symptoms compared to 27% in the beta-blocker users without diabetes (difference 95% CI: 4%-25%).

After multivariable adjustment (model 5, N = 684), the OR for the risk of depressive symptoms in patients with diabetes was 1.41 (95% CI: 1.00-1.98) and for beta-blocker users it was 1.12 (95% CI: 0.80-1.56) (Table 2). As expected, adding CVD to the full model (model 5) attenuated the association between diabetes or beta-blocker use and depressive symptoms.

**Table 2.** Logistic analysis for factors associated with depressive symptoms (BDI-II ⩾ 16, N = 290) among 684 chronic dialysis patients.

BDI ⩾ 16^a^	Crude		Model 1		Model 2		Model 3		Model 4		Model 5	
	OR	95% CI	OR	95% CI	OR	95% CI	OR	95% CI	OR	95% CI	OR	95% CI
Age, y	1.00	0.99-1.00	1.00	0.99-1.01	1.01	0.99-1.02	1.01	1.00-1.02	1.00	0.99-1.02	1.00	0.99-1.02
Sex, men	0.74	0.54-1.01	0.74	0.54-1.02	0.81	0.58-1.14	0.81	0.58-1.14	0.82	0.58-1.14	0.81	0.58-1.14
Education, high	0.71^b^	0.52-0.97	0.72^b^	0.52-0.99	0.77	0.55-1.07	0.77	0.55-1.08	0.81	0.58-1.14	0.81	0.57-1.14
Ethnicity, immigrant	1.65^b^	1.22-2.25	1.68^b^	1.21-2.34	1.79^b^	1.25-2.56	1.79^b^	1.25-2.56	1.67^b^	1.16-2.41	1.67^b^	1.16-2.41
Smoking, current	2.61^b^	1.81-3.77	2.59^b^	1.78-3.75	2.98^b^	2.03-4.39	2.97^b^	2.02-4.38	2.98^b^	2.02-4.39	2.96^b^	2.01-4.37
Alcohol use, yes	0.54^b^	0.37-0.78	0.57^b^	0.39-0.82	0.64^b^	0.43-0.95	0.65^b^	0.43-0.96	0.66^b^	0.44-0.98	0.66^b^	0.44-0.99
Dialysis vintage, mo	1.00	1.00-1.01	1.00	1.00-1.01	1.00	1.00-1.01	1.00	1.00-1.01	1.00	1.00-1.01	1.00	1.00-1.01
CVD, yes	1.20	0.88-1.62	1.29	0.94-1.78	1.20	0.86-1.68	1.21	0.86-1.68	1.12	0.79-1.58	1.12	0.79-1.58
Hypertension, yes	1.14	0.83-1.57	1.16	0.84-1.59	1.13	0.81-1.57	1.13	0.81-1.58	1.07	0.77-1.51	1.07	0.76-1.50
Diabetes, yes	1.54^b^	1.13-2.09	1.59^b^	1.16-2.17	1.42^b^	1.02-1.97	1.44^b^	1.03-2.01	1.44^b^	1.03-2.01	1.41	1.00-1.98
Beta-blocker use, yes	1.34	0.98-1.82	1.37^b^	1.01-1.87	1.19	0.86-1.65	1.19	0.86-1.65	1.14	0.82-1.58	1.12	0.80-1.56
Lipophilic beta-blocker use, yes	1.38^b^	1.01-1.87	1.41^b^	1.03-1.92	1.22	0.88-1.69	1.22	0.88-1.69	1.16	0.83-1.62	1.14	0.81-1.59

Abbreviations: 95% CI, 95% confidence interval; CVD, cardiovascular disease.

Model 1 = adjusted for age and sex. Model 2 = model 1 plus additional adjustment for smoking, alcohol consumption, ethnicity and educational level. Model 3 = model 2 plus additional adjustment for dialysis vintage. Model 4 = model 3 plus additional adjustment for diabetes (except in the model were the effect of diabetes on depression was studied). Model 5 = Model 4 plus additional adjustment for cardiovascular disease.

a On the basis of the Beck Depression Inventory (1979).

b *P* < .05.

### Relative excess risk of depressive symptoms due to interaction

After adjustment for potential confounders, we found an 1.7-fold increased risk of depressive symptoms in dialysis patients with diabetes who use beta-blockers compared to patients without diabetes and without using beta-blockers, indicating effect modification (Table 3). No increased risk of depressive symptoms was observed in dialysis patients without diabetes who use beta-blockers compared to patients without diabetes and without using beta-blockers (OR = 1.16, 95% CI: 0.76-1.77). To examine the joint and separate effects of the risk factors diabetes and beta-blocker use on depressive symptoms, the synergy index and RERI were calculated (Table 3). After adjustment for age, sex, ethnicity and CVD, this resulted in *S* = 1.87 (95% CI: 0.19-18.13) and RERI = 0.34 (95% CI: −0.75 to 1.43). This indicated that the joint effect of diabetes and beta-blocker use on depressive symptoms is larger than the separate effects. The same analysis was performed on lipophilic beta-blockers only, where this relation persisted and became stronger (Supplemental Table S1).

**Table 3.** Risk of beta-blockers and/or diabetes on depressive symptoms (BDI-II ⩾ 16, N = 290) among 684 chronic dialysis patients.

	Diabetes	Beta-blocker use	N	N BDI ⩾ 16^a^	Crude		Model 1		Model 2		Model 3	
					OR	95% CI	OR	95% CI	OR	95% CI	OR	95% CI
	No	No	196	71	1.00	(reference)	1.00	(reference)	1.00	(reference)	1.00	(reference)
	Yes	No	101	43	1.31	0.80-2.13	1.38	0.84-2.26	1.27	0.77-2.11	1.23	0.74-2.06
	No	Yes	197	78	1.15	0.77-1.74	1.20	0.80-1.81	1.19	0.79-1.80	1.16	0.76-1.77
	Yes	Yes	190	98	1.88^b^	1.25-2.82	1.97^b^	1.31-2.99	1.81^b^	1.19-2.75	1.73^b^	1.12-2.69
Total			684	290								
Synergy index					1.91	0.28-13.13	1.69	0.32-9.06	1.75	0.24-12.53	1.87	0.19-18.13
RERI					0.42	−0.68 to 1.51	0.40	−0.76 to 1.56	0.35	−0.75 to 1.44	0.34	−0.75 to 1.43

The reference group are patients without diabetes and not using beta-blockers. Crude model consists of the above mentioned factors and their effect on depressive symptoms. Model 1 = adjusted for age and sex. Model 2 = model 1 plus additional adjustment for ethnicity. Model 3 = model 2 plus additional adjustment for cardiovascular disease.

The amount of biologic interaction was calculated using the synergy index [*S* = (OR_++_ − 1)/((OR_+−_ − 1) + (OR_−+_ − 1))], which describes the ratio of the joint effect (presence of both risk factors) to the sum of the effects (presence of each risk factor in the absence of the other). When *S* = 1, there is no biologic interaction. Also the relative excess risk [RERI = OR_++_ − OR_+−_ − OR_−+_ + 1] was calculated. This approach looks if the effect when both risk factors are jointly presented differs compared to the sum of both risk factors presented separately. When RERI = 1, there is no biologic interaction.

Abbreviation: 95% CI, 95% confidence interval.

a On the basis of the Beck Depression Inventory (1979).

b *P* < 0.05.

### Sensitivity analyses

The effects became slightly stronger when using mean substitution according to the manual data (Supplemental Tables S2A and S2B) or complete cases (Supplemental Tables S3A and S3B).

## Discussion

This Dutch multi-ethnical cohort of chronic dialysis patients shows a prevalence of depressive symptoms of 37% and 26% in those with diabetes versus without diabetes, and 34% and 27% in beta-blocker users versus non-users, respectively. Chronic dialysis patients with diabetes had a 1.4-fold elevated risk of depressive symptoms compared to patients without diabetes. Use of beta-blockers, in addition to diabetes, substantially further increased this risk to 1.7-fold.

The overall prevalence of 31% of depressive symptoms in our cohort is in line with the study of Griva et al^34^ performed among dialysis patients. They showed a prevalence of depressive symptoms (defined as a BDI-II score ⩾16) of 42% among in hospital HD patients, 49% in continuous ambulatory PD, 26% in automated PD, and a much lower rate among home HD patients of 8%.^34^ To compare, the prevalence of depression in the general adult population is about 5%.^35^

In our cohort of chronic dialysis patients, we showed that the prevalence of depressive symptoms in patients with diabetes was 11% higher compared to those without diabetes (37% vs 26%). After multivariable adjustment, patients with diabetes had a 41% increased risk of depressive symptoms compared to patients without diabetes (OR 1.41, 95% CI: 1.00-1.98). These results are in line with the meta-analysis of Ali et al^36^ in 51 331 adults, where a higher prevalence of depressive symptoms was observed among people with diabetes versus without: 17.6% versus 9.8% (OR 1.6, 95% CI: 1.2-2.0). The co-occurrence of diabetes and depression might be ascribed to the psychosocial and psychological impact of diabetes, microvascular brain lesions caused by diabetes, a potential common genetic susceptibility or inflammation.^37^ The progression of kidney function decline combined with diabetes is presumably also associated with an increased risk and severity of depressive symptoms.^38,39^

After multivariable adjustment, beta-blocker users had a higher, but non-significant risk of depressive symptoms compared to non-users (OR 1.12, 95% CI: 0.80-1.56). A study performed by Agustini et al^13^ showed similar results. The use of beta-blockers was significantly associated with an increased prevalence of depressive symptoms in 14 000 hypertensive older adults without a history of CVD or heart failure. Lipophilic beta-blockers were stronger associated with increased prevalence of depressive symptoms, compared to hydrophilic beta-blockers.^13^ The Rotterdam Study, a prospective study of 5104 older adults in which participants with CVD were included (11% with post-MI and 4% with chronic heart failure), showed an association between the use of lipophilic beta-blockers and an increased risk of depressive symptoms.^40^ In the present study 375 (97%) patients used lipophilic beta-blockers. Lipophilic beta-blockers are able to pass the blood-brain-barrier and may therefore cause central nervous system side-effects, such as depressive symptoms and sleeping problems.^14,15^ Exclusion of Atenolol and Sotalol users, which display hydrophilic properties and are therefore suggested to be unable to cross the blood-brain-barrier, increased the risk of depressive symptoms in our analysis.

We found an almost 2-fold increased risk of depressive symptoms in chronic dialysis patients with diabetes and using beta-blockers compared to patients without diabetes and not using beta-blockers. This finding indicates effect modification, namely that diabetes and beta-blockers may cause depressive symptoms via separate pathophysiological mechanisms.

There are some limitations to this study. This study has an observational design, which does not allow us to make conclusions about the causality of the association. The broad 95% CI underscores the relative small sample size. The BDI-II is a validated tool for screening for depression, but it is not a formal diagnostic tool. Therefore, we refer to depressive symptoms and not depression. In addition, depression is a multicausal disorder and we only studied one possible factor, namely beta-blocker use in high risk patients with diabetes. Thereby, the primary indication, doses and adherence to beta-blocker use might interfere with the relation with depressive symptoms. Lipophilic beta-blockers, in particular propranolol, are often prescribed to treat anxiety.^41^ Therefor it is not surprising that propranolol is sometimes found to have a strong association with depression.^42^ Despite the fact that we did not include propranolol in our analyses, we cannot exclude confounding by indication for the other included beta-blockers.

In addition, no distinction was made between single prescription and long term use, which might influence the results. Despite the fact that we have corrected for multiple confounding factors, including CVD, again confounding by indication cannot be excluded by this study design.

Physicians should be aware of the possible neuropsychiatric adverse effects of beta-blockers among patients with diabetes on dialysis. Beta-blockers are frequently prescribed for primary and secondary prevention of CVD in dialysis patients, constituting 64% of all prescribed cardiovascular medications.^43^ On the other hand, undertreatment of CVD in patients with mood disorders still remains a serious concern. Clinicians should take into account the balance between mental health and quality of life, as well as possible gains in morbidity and mortality.

This study provides evidence for a possible association between lipophilic beta-blocker use and depressive symptoms in chronic dialysis patients, especially in those with diabetes. Use of lipophilic beta-blockers among dialysis patients with diabetes, was associated with an almost 2-fold increased risk of depressive symptoms. Lipophilic beta-blockers use was not materially associated with an increased risk of depressive symptoms in dialysis patients without diabetes.

## Supplemental Material

sj-docx-1-end-10.1177_11795514221119446 – Supplemental material for Association Between Lipophilic Beta-Blockers and Depression in Diabetic Patients on Chronic Dialysis Click here for additional data file. Supplemental material, sj-docx-1-end-10.1177_11795514221119446 for Association Between Lipophilic Beta-Blockers and Depression in Diabetic Patients on Chronic Dialysis by Robin Lengton, Robbert W Schouten, Els Nadort, Elisabeth FC van Rossum, Friedo W Dekker, Carl EH Siegert and Ellen K Hoogeveen in Clinical Medicine Insights: Endocrinology and Diabetes

## Declarations

**Ethics Approval and Consent to Participate:** The DIVERS-I study was conducted in accordance with the Declaration of Helsinki and was approved by the medical ethics committee of the VU university medical centre (approval number: 2010/064). Informed consent was obtained from all individual participants included in the study.

**Consent for Publication:** Not applicable.

**Availability of Data and Materials:** As our data could be used to identify individuals, privacy concerns prevent us from allowing them to be publically available. Nonetheless, data will be available (conditional on agreement on privacy matters and appropriate usage of the data) upon request from the principal investigator (Carl E.H. Siegert, E-mail: c.siegert@olvg.nl).
