# Supplementary material for: Association Between Lipophilic Beta-Blockers and Depression in Diabetic Patients on Chronic Dialysis
Source: Clin Med Insights Endocrinol Diabetes. 2022 Aug 29;15:11795514221119446. doi: 10.1177/11795514221119446 (PMC9434677; doi:10.1177/11795514221119446)
Supplement: sj-docx-1-end-10.1177_11795514221119446 – Supplemental material for Association Between Lipophilic Beta-Blockers and Depression in Diabetic Patients on Chronic Dialysis [file sj-docx-1-end-10.1177_11795514221119446.docx]

SUPPLEMENTARY

|  |  |  |  |  | Crude | | Model 1 | | Model 2 | | Model 3 | |
| --- | --- | --- | --- | --- | --- | --- | --- | --- | --- | --- | --- | --- |
|  | **Diabetes** | **Beta-blocker use** | **N** | **N**  **BDI≥16^a^** | **OR** | **95% CI** | **OR** | **95% CI** | **OR** | **95% CI** | **OR** | **95% CI** |
|  | No | No | 196 | 71 | 1.00 | (reference) | 1.00 | (reference) | 1.00 | (reference) | 1.00 | (reference) |
|  | Yes | No | 101 | 43 | 1.31 | 0.80 to 2.13 | 1.37 | 0.84 to 2.26 | 1.28 | 0.77 to 2.11 | 1.23 | 0.73 to 2.05 |
|  | No | Yes | 188 | 75 | 1.17 | 0.77 to 1.77 | 1.21 | 0.80 to 1.84 | 1.20 | 0.79 to 1.82 | 1.16 | 0.76 to 1.77 |
|  | Yes | Yes | 187 | 98 | 1.94^b^ | 1.29 to 2.92 | 2.03^b^ | 1.34 to 3.08 | 1.87^b^ | 1.23 to 2.85 | 1.77^b^ | 1.14 to 2.74 |
| Total |  |  | 672 | 287 |  |  |  |  |  |  |  |  |
| Synergy index |  |  |  |  | 1.98 | 0.30 to 13.00 | 1.76 | 0.34 to 9.23 | 1.82 | 0.27 to 12.52 | 2.00 | 0.20 to 20.02 |
| RERI |  |  |  |  | 0.47 | -0.65 to 1.58 | 0.45 | -0.74 to 1.63 | 0.39 | -0.73 to 1.51 | 0.38 | -0.71 to 1.48 |

**Table S1. Sensitivity analysis on multiple imputation data, risk of lipophilic beta-blockers and/or diabetes on depressive symptoms (BDI-II ≥ 16, N=287)** **among 672 chronic dialysis patients.**

95% CI, 95% confidence interval.
^a^On the basis of the Beck Depression Inventory (1979).
^b^P < 0.05.
The reference group are patients without diabetes and not using beta-blockers. Crude model consists of the above mentioned factors and their effect on depressive symptoms. Model 1 = adjusted for age and sex. Model 2 = model 1 plus additional adjustment for ethnicity. Model 3 = model 2 plus additional adjustment for cardiovascular disease.

The amount of interaction was calculated using the synergy index [S=(OR_++_ – 1) / ((OR_+-_ – 1) + (OR_-+_ – 1))], which describes the ratio of the joint effect (presence of both risk factors) to the sum of the effects (presence of each risk factor in the absence of the other). When S=1, there is no biologic interaction. Also the relative excess risk [RERI= OR_++_ - OR_+-_ - OR_-+_ + 1] was calculated. This approach looks if the effect when both risk factors are jointly presented differs compared to the sum of both risk factors presented separately. When RERI=1, there is no biologic interaction.

**Table S2A. Sensitivity analysis on mean substitution data according to the manual, logistic analysis for factors associated with depressive symptoms (BDI-II ≥ 16, N=185)** **among 603 chronic dialysis patients.**

| BDI≥16^a^ | Crude | |  | Model 1 | |  | Model 2 | |  | Model 3 | |  | Model 4 | | Model 5 | |
| --- | --- | --- | --- | --- | --- | --- | --- | --- | --- | --- | --- | --- | --- | --- | --- | --- |
|  | OR | 95% CI |  | OR | 95% CI |  | OR | 95% CI |  | OR | 95% CI |  | OR | 95% CI | OR | 95% CI |
| Age, yr | 0.99 | 0.98 to 1.01 |  | 0.99 | 0.98 to 1.01 |  | 1.00 | 0.98 to 1.01 |  | 1.00 | 0.98 to 1.01 |  | 1.00 | 0.98 to 1.01 | 0.99 | 0.98 to 1.01 |
| Sex, men | 1.03 | 0.73 to 1.45 |  | 1.05 | 0.74 to 1.49 |  | 1.16 | 0.77 to 1.75 |  | 1.16 | 0.77 to 1.76 |  | 1.17 | 0.77 to 1.77 | 1.18 | 0.76 to 1.78 |
| Education, high | 0.51^b^ | 0.35 to 0.74 |  | 0.47^b^ | 0.32 to 0.70 |  | 0.50^b^ | 0.33 to 0.76 |  | 0.51^b^ | 0.34 to 0.77 |  | 0.54^b^ | 0.35 to 0.81 | 0.53^b^ | 0.35 to 0.81 |
| Ethnicity, immigrant | 2.32^b^ | 1.62 to 3.32 |  | 2.38^b^ | 1.62 to 3.48 |  | 1.93^b^ | 1.25 to 2.97 |  | 1.93^b^ | 1.26 to 2.97 |  | 1.77^b^ | 1.14 to 2.74 | 1.76^b^ | 1.14 to 2.73 |
| Smoking, current | 1.47 | 0.95 to 2.28 |  | 1.42 | 0.91 to 2.21 |  | 1.48 | 0.91 to 2.39 |  | 1.46 | 0.90 to 2.37 |  | 1.51 | 0.93 to 2.45 | 1.49 | 0.92 to 2.43 |
| Alcohol use, yes | 0.57^b^ | 0.37 to 0.86 |  | 0.58^b^ | 0.37 to 0.89 |  | 0.81 | 0.50 to 1.32 |  | 0.81 | 0.50 to 1.33 |  | 0.84 | 0.51 to 1.38 | 0.84 | 0.51 to 1.38 |
| Dialysis vintage, months | 1.00 | 1.00 to 1.01 |  | 1.00 | 1.00 to 1.01 |  | 1.00 | 1.00 to 1.01 |  | 1.00 | 1.00 to 1.01 |  | 1.00 | 1.00 to 1.01 | 1.00 | 1.00 to 1.01 |
| CVD, yes | 1.38 | 0.98 to 1.93 |  | 1.54^b^ | 1.07 to 2.20 |  | 1.22 | 0.82 to 1.83 |  | 1.22 | 0.82 to 1.82 |  | 1.08 | 0.71 to 1.63 | 1.08 | 0.71 to 1.63 |
| Hypertension, yes | 1.10 | 0.78 to 1.57 |  | 1.11 | 0.78 to 1.58 |  | 1.13 | 0.75 to 1.68 |  | 1.12 | 0.75 to 1.67 |  | 1.04 | 0.69 to 1.56 | 1.04 | 0.69 to 1.56 |
| Diabetes, yes | 1.79^b^ | 1.27 to 2.51 |  | 1.91^b^ | 1.35 to 2.71 |  | 1.69^b^ | 1.14 to 2.52 |  | 1.70^b^ | 1.14 to 2.53 |  | 1.70^b^ | 1.14 to 2.53 | 1.67^b^ | 1.11 to 2.52 |
| Beta-blocker use, yes | 1.29 | 0.91 to 1.82 |  | 1.29 | 0.91 to 1.82 |  | 1.42 | 0.96 to 2.10 |  | 1.42 | 0.96 to 2.09 |  | 1.31 | 0.88 to 1.96 | 1.31 | 0.87 to 1.96 |
| Lipophilic beta-blocker use, yes | 1.31 | 0.93 to 1.86 |  | 1.31 | 0.92 to 1.85 |  | 1.45 | 0.98 to 2.15 |  | 1.44 | 0.97 to 2.14 |  | 1.33 | 0.89 to 1.99 | 1.33 | 0.89 to 2.00 |

95% CI, 95% confidence interval; CVD, cardiovascular disease.  ^a^On the basis of the Beck Depression Inventory (1979).
^b^P < 0.05.
Model 1 = adjusted for age and sex. Model 2 = model 1 plus additional adjustment for smoking, alcohol consumption, ethnicity and educational level. Model 3 = model 2 plus additional adjustment for dialysis vintage. Model 4 = model 3 plus additional adjustment for diabetes, except in the model were the effect of diabetes on depression was studied. Model 5 = Model 4 plus additional adjustment for cardiovascular disease.

**Table S2B. Sensitivity analysis on mean substitution data according to the manual, risk of beta-blockers and/or diabetes on depressive symptoms (BDI-II ≥ 16, N=185)** **among 603 chronic dialysis patients.**

|  |  |  |  |  | Crude | | Model 1 | |
| --- | --- | --- | --- | --- | --- | --- | --- | --- |
|  | **Diabetes** | **Beta-blocker use** | **N** | **N**  **BDI≥16^a^** | **OR** | **95% CI** | **OR** | **95% CI** |
|  | No | No | 179 | 45 | 1.00 | (reference) | 1.00 | (reference) |
|  | Yes | No | 90 | 27 | 1.22 | 0.71 to 2.13 | 1.31 | 0.75 to 2.30 |
|  | No | Yes | 166 | 42 | 0.91 | 0.57 to 1.46 | 0.90 | 0.56 to 1.46 |
|  | Yes | Yes | 168 | 71 | 2.00^b^ | 1.28 to 3.12 | 2.11^b^ | 1.35 to 3.32 |
| Total |  |  | 603 | 185 |  |  |  |  |
| Synergy index |  |  |  |  | 7.53 | 0.02 to 3522.22 | 5.25 | 0.08 to 327.75 |
| RERI |  |  |  |  | 0.87 | -0.31 to 2.05 | 0.90 | -0.36 to 2.16 |

95% CI, 95% confidence interval.
^a^On the basis of the Beck Depression Inventory (1979).
^b^P < 0.05.
The reference group are patients without diabetes and not using beta-blockers. Crude model consists of the above mentioned factors and their effect on depressive symptoms. Model 1 = adjusted for age and sex.

The amount of interaction was calculated using the synergy index [S=(OR_++_ – 1) / ((OR_+-_ – 1) + (OR_-+_ – 1))], which describes the ratio of the joint effect (presence of both risk factors) to the sum of the effects (presence of each risk factor in the absence of the other). When S=1, there is no biologic interaction. Also the relative excess risk [RERI= OR_++_ - OR_+-_ - OR_-+_ + 1] was calculated. This approach looks if the effect when both risk factors are jointly presented differs compared to the sum of both risk factors presented separately. When RERI=1, there is no biologic interaction.

**Table S3A. Sensitivity analysis on complete cases, logistic analysis for factors associated with depressive symptoms (BDI-II ≥ 16, N=163)** **among 533 chronic dialysis patients.**

| BDI≥16^a^ | Crude | |  | Model 1 | |  | Model 2 | |  | Model 3 | |  | Model 4 | | Model 5 | |
| --- | --- | --- | --- | --- | --- | --- | --- | --- | --- | --- | --- | --- | --- | --- | --- | --- |
|  | OR | 95% CI |  | OR | 95% CI |  | OR | 95% CI |  | OR | 95% CI |  | OR | 95% CI | OR | 95% CI |
| Age, yr | 0.99 | 0.98 to 1.01 |  | 0.99 | 0.98 to 1.01 |  | 1.00 | 0.99 to 1.02 |  | 1.00 | 0.99 to 1.02 |  | 1.00 | 0.98 to 1.01 | 1.00 | 0.98 to 1.01 |
| Sex, men | 0.91 | 0.62 to 1.34 |  | 0.94 | 0.64 to 1.38 |  | 1.04 | 0.67 to 1.62 |  | 1.04 | 0.67 to 1.62 |  | 1.05 | 0.68 to 1.64 | 1.05 | 0.68 to 1.64 |
| Education, high | 0.56^b^ | 0.38 to 0.82 |  | 0.53^b^ | 0.36 to 0.80 |  | 0.57^b^ | 0.37 to 0.87 |  | 0.57^b^ | 0.37 to 0.87 |  | 0.59^b^ | 0.39 to 0.91 | 0.59^b^ | 0.39 to 0.91 |
| Ethnicity, immigrant | 2.36^b^ | 1.61 to 3.48 |  | 2.42^b^ | 1.60 to 3.65 |  | 2.06^b^ | 1.30 to 3.24 |  | 2.07^b^ | 1.31 to 3.27 |  | 1.92^b^ | 1.21 to 3.06 | 1.92^b^ | 1.21 to 3.06 |
| Smoking, current | 1.53 | 0.97 to 2.41 |  | 1.48 | 0.93 to 2.35 |  | 1.56 | 0.95 to 2.58 |  | 1.57 | 0.95 to 2.59 |  | 1.59 | 0.96 to 2.63 | 1.59 | 0.96 to 2.65 |
| Alcohol use, yes | 0.60^b^ | 0.38 to 0.92 |  | 0.61^b^ | 0.39 to 0.96 |  | 0.87 | 0.52 to 1.43 |  | 0.86 | 0.52 to 1.42 |  | 0.89 | 0.53 to 1.48 | 0.89 | 0.53 to 1.48 |
| Dialysis vintage, months | 1.00 | 1.00 to 1.01 |  | 1.00 | 1.00 to 1.01 |  | 1.00 | 1.00 to 1.00 |  | 1.00 | 1.00 to 1.00 |  | 1.00 | 1.00 to 1.00 | 1.00 | 1.00 to 1.00 |
| CVD, yes | 1.22 | 0.84 to 1.77 |  | 1.35 | 0.91 to 1.99 |  | 1.09 | 0.71 to 1.66 |  | 1.08 | 0.71 to 1.65 |  | 0.97 | 0.63 to 1.50 | 0.97 | 0.63 to 1.50 |
| Hypertension, yes | 1.14 | 0.78 to 1.67 |  | 1.15 | 0.78 to 1.68 |  | 1.14 | 0.75 to 1.74 |  | 1.13 | 0.74 to 1.73 |  | 1.06 | 0.69 to 1.63 | 1.06 | 0.69 to 1.63 |
| Diabetes, yes | 1.64^b^ | 1.13 to 2.39 |  | 1.75^b^ | 1.19 to 2.55 |  | 1.58^b^ | 1.04 to 2.39 |  | 1.56^b^ | 1.03 to 2.37 |  | 1.56^b^ | 1.03 to 2.37 | 1.57^b^ | 1.02 to 2.42 |
| Beta-blocker use, yes | 1.40 | 0.96 to 2.04 |  | 1.40 | 0.96 to 2.05 |  | 1.35 | 0.89 to 2.04 |  | 1.34 | 0.88 to 2.03 |  | 1.24 | 0.81 to 1.90 | 1.25 | 0.82 to 1.93 |
| Lipophilic beta-blocker use, yes | 1.44 | 0.99 to 2.11 |  | 1.44 | 0.99 to 2.11 |  | 1.39 | 0.92 to 2.11 |  | 1.38 | 0.91 to 2.10 |  | 1.29 | 0.84 to 1.97 | 1.30 | 0.84 to 2.00 |

95% CI, 95% confidence interval; CVD, cardiovascular disease.  ^a^On the basis of the Beck Depression Inventory (1979).
^b^P < 0.05.
Model 1 = adjusted for age and sex. Model 2 = model 1 plus additional adjustment for smoking, alcohol consumption, ethnicity and educational level. Model 3 = model 2 plus additional adjustment for dialysis vintage. Model 4 = model 3 plus additional adjustment for diabetes, except in the model were the effect of diabetes on depression was studied. Model 5 = Model 4 plus additional adjustment for cardiovascular disease.

**Table S3B. Sensitivity analysis on complete cases, risk of beta-blockers and/or diabetes on depressive symptoms (BDI-II ≥ 16, N=163)** **among 533 chronic dialysis patients.**

|  |  |  |  |  | Crude | | Model 1 | |
| --- | --- | --- | --- | --- | --- | --- | --- | --- |
|  | **Diabetes** | **Beta-blocker use** | **N**  **Total** | **N**  **BDI≥16^a^** | **OR** | **95% CI** | **OR** | **95% CI** |
|  | No | No | 160 | 41 | 1.00 | (reference) | 1.00 | (reference) |
|  | Yes | No | 73 | 21 | 1.17 | 0.63 to 2.18 | 1.24 | 0.67 to 2.32 |
|  | No | Yes | 150 | 40 | 1.06 | 0.64 to 1.75 | 1.04 | 0.63 to 1.74 |
|  | Yes | Yes | 150 | 61 | 1.99^b^ | 1.23 to 3.22 | 2.09^b^ | 1.28 to 3.40 |
| Total |  |  | 533 | 163 |  |  |  |  |
| Synergy index |  |  |  |  | 4.36 | 0.07 to 265.0 | 3.80 | 0.13 to 112.90 |
| RERI |  |  |  |  | 0.76 | -0.53 to 2.06 | 0.80 | -0.55 to 2.16 |

95% CI, 95% confidence interval.
^a^On the basis of the Beck Depression Inventory (1979).
^b^P < 0.05.
The reference group are patients without diabetes and not using beta-blockers. Crude model consists of the above mentioned factors and their effect on depressive symptoms. Model 1 = adjusted for age and sex.

The amount of interaction was calculated using the synergy index [S=(OR_++_ – 1) / ((OR_+-_ – 1) + (OR_-+_ – 1))], which describes the ratio of the joint effect (presence of both risk factors) to the sum of the effects (presence of each risk factor in the absence of the other). When S=1, there is no biologic interaction. Also the relative excess risk [RERI= OR_++_ - OR_+-_ - OR_-+_ + 1] was calculated. This approach looks if the effect when both risk factors are jointly presented differs compared to the sum of both risk factors presented separately. When RERI=1, there is no biologic interaction.
